# Explicating practice norms and tensions between values in resident training in family medicine

**DOI:** 10.1186/s12875-020-01242-6

**Published:** 2020-08-25

**Authors:** Morhaf Al Achkar

**Affiliations:** Department of Family Medicine, University of Washington, Seattle, WA USA

**Keywords:** Reflective practice, Video-reviews, Peer-learning, Resident education

## Abstract

**Background:**

Residency programs have the intricate and complex role of training health care providers. But little is known about what residents and attendings consider norms of practice or the tensions among different values residents are expected to uphold. Thus, dialogical and reflective frameworks are being explored for resident learning.

**Methods:**

This study examined the use of facilitated conversations with groups of residents and attending physicians while reviewing video-recorded resident–patient interactions. The conversations were recorded, transcribed, and qualitatively analyzed.

**Results:**

A total of 24 residents and 10 attendings participated in conversations while separately and in parallel groups reviewing 15 resident sessions. Residents explicated the norms of practice and evaluated their performance, which often agreed with those of attending physicians in calling out important learning opportunities. When disagreement occurred, residents’ explications of their reasoning were often relevant and, via reflection and dialogue, helped clarify intentions that were not apparent in the videos. Residents and attendings often judged actions on more than one domain of value. For instance, if a resident addressed problems, built relationships in a timely manner, and acted autonomously without jeopardizing the quality of care, she satisfactorily performed her duty.

**Conclusions:**

Practice norms and value struggles were addressed by participants during reviews, which provided a promising framework for learning and assessment. Also, the non-hierarchical structure opened space to acknowledge a diversity of positions and for tensions among values to be explicated.

## Background

The complex role of training doctors is ascribed to residency programs, which must support resident learning while still providing safe patient care. Residencies grapple with strategies that can simultaneously uphold the two ends [1–6].

An increasing body of evidence supports the premise that resident engagement in reflection and self-assessment improves learning [7–11]. There is also evidence that learning from peers may be equal to or more effective than learning from an attending [12]. Furthermore, many viable learning models exist that center on learners, with non-hierarchical engagement and empowerment of participants [13–17].

In contrast, learning has historically been viewed as adaptation, where the learner performs a behavior, then an evaluator gives feedback, and finally, the learner performs again [7, 8, 18]. In this style of assessment, an evaluator observes an object and judges its behavior. Variations exist around who is performing the evaluation (e.g., attending, peer, self) and who is giving the feedback (attending or peer) [7, 8, 19–21].

Within the meta-theoretical framework of subject–object relations, self-assessment has been considered a “problem child,” and little is understood about its workings. Despite mounting evidence that self-evaluation enhances learning [7, 8], it has often been criticized as inaccurate, unreliable, and “biased.” [22–25]

While there is a large body of literature on “best practices” from experts’ perspectives and on checklists used for judging behaviors from the perspective of observers, there is little known about the perspectives of participants in dialogical and reflective learning. This study helps fill the knowledge gap through 1) examining resident and attending views on norms of practice for primary care functions, and 2) demonstrating the tensions between values residents are expected to uphold while developing competency.

Achieving these aims helps in developing an inclusive and reflective dialogues, learner-learner and learner–educator, about resident competency and learning in clinical settings.

## Methods

I used a critical qualitative structure for this study. Qualitative research investigates human phenomena that, by their nature, do not lend themselves to quantitative methods. A qualitative method is fitting because it allows for the inclusion of multiple perspectives (e.g., resident, faculty, and researcher). The word “critical” indicate concerns with social theory and an awareness of social structure, culture, power, and human agency in this inquiry [26]. The study was reviewed and granted an exemption by the Indiana University IRB.

### Setting

This study was conducted at a university-based family medicine residency program where residents and attending physicians provide full-scope family medical services through an outpatient clinic located in the heart of a metropolitan area. The Accreditation Council for Graduate Medical Education requires that residency training includes observation of resident interactions with patients. To that end, this residency implemented video reviews. Between August and November 2016, 31 resident–patient interactions were recorded. These video recordings were first reviewed by groups of two to four faculty members and then by groups of three residents, one from each year of training. The review sessions lasted approximately 2 h, during which the group watched three videos, one from each resident. The separate interactions among faculty members and among the residents were all audiotaped.

### Participants

All residents and faculty at the residency program were included in the video review experience since it was conducted for educational purposes. For each resident, one to three videos were recorded, one of which was selected for review based on the quality of the recording. Residents were informed on the day they would be video-recorded. Only adult clinic patients with chronic or acute medical conditions who spoke English and were willing to be video-recorded were included.

I used liberal inclusion criteria for the patients to assure findings could be generalized to other clinical settings and specialties. I excluded video recordings that were extremely short (< 7 min) and those with poor visual or sound quality. I am a male attending physician, moderated both the attending group and resident group discussions. A psychologist provider co-facilitated some sessions to bring sensitivity to behavioral health matters. I was interested in this work to ensure adequate supervision of residents and to provide open space for learning and conversations. To provide separation between the educational role of this tool and the research, I conducted the data analysis independently and after moving to another institute.

For the purposes of this study and to ensure saturation, I selected 15 residents from the population of 31 reviewed residents. This was a purposive sampling. For a resident to be included, recordings from both the attending physicians’ sessions and the residents’ sessions had to be available and of good quality in terms of content and duration. Further, I aimed to include a diversity of clinical cases and a similar number of residents from each class. Each video review took between 30 and 45 min.

### Data collection

Interactions between residents or faculty during the respective review sessions were audio recorded, and each session followed the same pattern. First, videos were presented in two- to three-minute segments before pausing for discussion, which was moderated using opening questions or specific prompts. Examples of opening questions are “What do you see?” “What do you think?” and “What else could or should be done?” Examples of prompts include “How was the health condition managed?” and “What else could or should be done to manage this health condition?” The MD moderator and psychologist co-facilitator debriefed after sessions to refine future interactions. All review session recordings were transcribed verbatim.

### Analysis

The analysis was completed by MA using the systematic strategy of critical theory-based qualitative methods outlined by Carspecken [26], which includes five steps: 1) Writing low-level codes: These are word-tags listed to mark segments of the text and required little abstraction, so they stay close to the primary records. 2) Constructing meaning fields: This step includes articulating the possible meanings inferred from statements made by the participants. The field often consists of statements separated by “and” or “or.” 3) Developing a validity reconstruction: This reconstruction involves explicating meanings that can be understood and described in terms of validity claims. Validity claims are statements or utterances that are criticizable in the sense that they need reasons to be justified. They, in turn, can serve as reasons for other claims. This step clarifies assumptions and makes explicit presumed norms (i.e., normative claims) and positions about truth (i.e., objective claims) as well as feelings and intentions (i.e., subjective claims). 4) Organizing themes: This step involves an iterative, inductive and deductive process. In this process, coded texts that included assessment of the adequacy of the performance were extracted. Utterances were thematically organized according to the topic or function they relate to (e.g., history taking, managing problem, using computer etc.). A hierarchical theme organization was developed to highlight what is considered right or appropriate and, on the other hand, what the participant judged a error or inappropriate. 5) Write up: findings were organized to reflect how a specific function ought to be done and, on the other hand, to present a reconstruction of what needs to be balanced in terms of contradicting values.

I used Microsoft Word for coding and Microsoft Excel for the thematic organization. To enhance the study’s trustworthiness, I used low inference in coding to maintain proximity between the content and the themes of analysis. I also practiced reflexivity by writing memos on the positions of teacher and doctor and by discussing the interpretations with peers and advisors. I have a master science in clinical research and did this study as part of his Ph.D. dissertation under the supervision of qualitative research advisors. Advisors and peers provided comments in person, over video-conference, and in written formats. These comments were used to refine the analysis.

## Results

The 15 resident sessions that were reviewed included (5 female residents; 5 residents from each class year). A total of 24 residents (8 females; 8 from each class) and 10 attendings (6 females; 7 MDs, 2 psychologists, and 1 nurse practitioner) participated in the conversations and were included in the analysis.

### Norms of practice regarding essential functions of a visit

From the perspectives of both attendings and residents, there are six critical norms of practice expected of residents:

1) *Residents should prepare for the visit before entering the room.* The participants valued knowing that a resident had reviewed charts and the paper slip that patients completed before the appointment. One attending commented, “*I think it was good that he referenced the stuff the patient had already filled out. I think that lets the patient know that he’s taken time to review her stuff.*” (A6) It was found unacceptable when residents appeared to be unaware of why a patient was there or only guessed at the reason. One resident reflected, “*I went to the computer and was trying to [look up] more information. I was thinking maybe I should have been more prepared before I stepped in.*” (R2) On the other hand, some residents gave insight that with a packed schedule, being prepared can be difficult. One resident explained, “s*ometimes it’s tough to look up 20 patients and know everything*.” (R14).

2) *Negotiating the agenda for the visit is recommended.* The participants valued residents starting a visit by setting the agenda, which can be done by illustrating and clarifying reasons for the appointment. An attending noted, “*He’s done pretty well setting an agenda for the visit. He was finding out if the patient has any concerns and then setting a plan.*” (A9) Residents noticed that when there was no clear agenda, the visit would go out of control. A resident reflected, “*[Five minutes into the visit] I was noticing that I didn’t have a set agenda. So, it needed to happen then since it wasn’t flowing naturally.*” (R7).

3) *Eliciting sufficient patient history is essential.* Regarding the content of history taking, the participants valued when residents gathered necessary information by asking the right questions. Taking a good history can mean screening for symptoms and risk factors, especially those associated with serious consequences or mental illnesses, which are often overlooked. It can also mean asking about social history and medications. Residents and attendings both pointed out suboptimal practices and omissions. For example, a patient’s suicidality was overlooked, and an attending remarked, “*If somebody has a [Patient Health Questionnaire] PHQ of 25 you have to ask, ‘Do you want to hurt yourself? Do you want to kill yourself or anybody else?’”* (A4) Regarding this incident, the resident reflected, “*[I should have obtained] more information and gotten a full history. I had no idea she was suicidal. I completely glanced over the PHQ9. I didn’t catch that.*” (R14).

Participants thought it was not useful to ask open-ended questions with patients who are not linear. On the other end of the spectrum, they also found it inappropriate to fail to ask for more explanation or assess symptoms in a closed-ended style when little information is gleaned. Attendings and residents frequently agreed on their assessments. For example, an attending once remarked, “*I think [the resident] is patient, I probably would not ask any open-ended questions. I’d ask yes/no type questions, especially when she starts off on those tangents.*” (A1) During the resident review session, the resident recognized that he could have done it differently, “*I think I got all the information that I wanted, but it took a little while. I could have potentially just kept asking/redirecting questions.*” (R5).

4) *Patients should have proper examinations.* The participants valued a resident telling patients what to expect before the exam. They expected a systematic and thorough exam with attention to aspects that would be relevant to the problem at hand. They also appreciated when findings were conveyed to the patient. One peer resident remarked, “*She explained what she was doing in real-time. She initially started with medical lingo, but then broke it down a bit to more layman’s terms.*” (R13).

Both residents and attendings paid attention to inappropriate examination techniques, and attendings were particularly concerned about insufficient exams or lack of diligence. An attending pointed out, “*Probably not getting nose to nose with the patient when you do your eye exam. He checked her left eye with his right eye!*” (A4).

5) *Making the diagnosis correctly is of primary importance.* The participants expressed satisfaction when residents considered differential diagnoses. Residents were particularly impressed when a peer had recognized the root of the problem despite a poorly presented patient story. All participants criticized relying solely on history without an adequate physical exam. They also called out incorrect diagnoses. Regarding one incident where a finger fracture healed poorly, the attending said, “*If the patient comes in to us and the fracture’s not healing, I’m not thinking diabetes. (Another attending:) I’m thinking, ‘Where’s your splint?!’”* (A9&A7) During the resident session, the resident shared about his thought process, “*I did the x-ray, and it was a month out, and he still, it wasn’t healing, so I thought maybe he had diabetes.”* (R9) Not recognizing the right etiology of the problem made this resident give inappropriate advice, “*They gave him a finger splint, so I told him to use it at work to prevent further injury and take it off after work to try exercising it a little bit!*” (R9).

Residents and attendings did not always maintain an agreement, however. For example, while attendings criticized a resident not being thorough, a resident mentioned reliance on gestalt feelings and considered that appropriate. The attending exclaimed, “*He sort of nailed in on carpal tunnel very fast. It’s almost as if he had the diagnosis in his mind and was trying to confirm it with the questions.*” (A1) But, during the resident review session, the involved resident explained, “*I was going with more of a gestalt feeling about it as opposed to following history, physical, etc. It doesn’t sound like there was any traumatic history. It doesn’t sound like there was any compromise in terms of function, no ER visits, no x-rays, nothing that was red flags to me at that point. I felt comfortable doing that.*” (R10).

*6)* *Carrying out proper management of health conditions is paramount.* The participants expected residents to prescribe the right medicine, develop a targeted plan, and do whatever else was needed, including providing information and counseling patients appropriately. Participants noted that residents should make their thoughts explicit to patients. They also should ensure that communication is at levels appropriate for the patient’s educational attainment. For example, an attending remarked, “*It was good that she used specific instructions and wrote them down for the family.*” (A9) Regarding the same interaction, a peer resident commented, “*The counseling was good. It was specific and giving clear instructions at the appropriate educational level.*” (R8).

The participants criticized when residents did not thoroughly address problems, gave hurried or inaccurate recommendations, or developed plans without measures to ensure follow-through. They also considered a resident’s avoidance of complicated health problems unacceptable. One resident reflected, “He has *a panic attack, but I don’t really want to address that, because he’s not going to get benzos from me. That’s what was going through my mind. ‘Let’s just try and glaze over this.’ I recognized it as a panic attack, but I also recognized it in my head that that would warrant benzos, which I don’t want to give him. I wanted to gloss over that and say, ‘Are you still seeing your counselor? Good. She’ll take care of that.’ Which isn’t good.*” (R6).

Attendings further criticized residents for failing to use specific communication strategies to convey recommendations. However, the reviewed residents were occasionally able to justify not using a particular approach within the context of the interactions. For example, an attending exclaimed, “*It would be helpful to have the patient use teach-back, ‘Can you tell me what the top three things are that I’ve asked you to do to take care of this wound?’”* (A3) But, the resident provided contradictory, yet valuable insight when he said, *“The patient was finishing my sentences. That’s how I knew, ‘Okay, he knows what I’m saying.’”* (R3).

### Tensions caused by differing values during a visit

The analysis identified five themes related to tensions between differing values.

1) *Addressing problems while building relationships.* The participants judged visits based on the success of addressing problems while simultaneously building a relationship as a goal in and of itself. Attendings considered that residents did well if they spent time engaging compassionately to build the relationship. At times, residents gave explanations on why they focused on problem-solving. One attending remarked, “*In a patient you’ve never seen before, you’re trying to build the relationship from the start. It would be helpful if you were showing that you were engaged and passionate about what’s going on with the patient.*” (A9) Regarding the same patient interaction, the reviewed resident that their focus on problems was a way to build rapport. They commented, “*I felt that it was important to address that problem being that it’s my first visit with her. I felt that if I focused on the pregnancy and were not addressing her complaint, I probably wouldn’t be doing justice to her. Also, in terms of building that rapport, it would probably reflect poorly on that.*” (R10).

2) *Performing duties within a reasonable time frame.* Participants considered residents to be doing a good job if they dealt with multiple problems or built rapport in a short period of time. They recognized the potential conflict between time constraints and building rapport. Participants criticized time-consuming habits such as using pen and paper for notes or attempting to address all problems in one visit. Agreements between the two sides were often the case. Regarding one visit, an attending commented, “*It’s just a lot longer than it needs to be. It was an hour by the time he went to talk to a preceptor.*” (A4) The reviewed resident had a similar position when they saw their video, “*I completely agree with [my peer]. The stuff I spent 10 minutes talking about, I could have probably done three minutes. I can improve on that.*” (R15).

3) *Completing tasks without compromising conversation.* The participants extensively discussed the use of computers to complete tasks in exam rooms. They looked positively on residents who asked for a patient’s permission before using the computer. Residents who are adept in this area maintain eye contact and sit facing patients, preferably at eye level. While they complete visit-related tasks on the computer, they maintain a genuine conversation, evidenced by reassuring sounds, paraphrasing, using reflection, and using teach-back, among other techniques. Participants agreed that computers could be particularly helpful for sharing results such as X-rays with patients or for obtaining information, such as medication dosages. However, they criticized when residents stared at the computer, acted in a hurry, or took detailed notes rather than brief reminders, especially when patients shared intimate information. For example, one attending commented, “*It feels like the visit is not focused on her as much. He is on his computer; ‘I am trying to get my notes done,’ that’s the impression I got.”* (A8) In a separate session, the reviewed resident remarked, *“I’m wondering, ‘What am I looking at?’ All the time, I’m looking on the screen. Yeah, I look at it too much.*” (R5).

4) *Supporting patient autonomy while asserting boundaries.* The participants accepted that residents can lay the groundwork for expectations and set boundaries. Residents can practice medicine only within the limits of their values and beliefs. They noted that residents could respectfully say no to prescribing certain medications, such as birth control methods based on religious views, or controlled substances if their use was deemed inappropriate. Here is one attending observing, “*He may be trying to lay all this groundwork so he can say, ‘This is why I’m not going to give you what you want.’ The patient’s using drugs.”* (A4) Similarly, a peer remarked on the same encounter that his peer, “*was setting boundaries.*”(R7) At the same time, residents should support patients in making appropriate health behavior (e.g., smoking cessation) changes when the patient is ready. Participants felt that patients should be given options to choose from, so that doctors can identify their true preferences. Here is one peer resident commenting, “*She figured out the patient preference and gave her all the options.*” (R1).

5) *Acting autonomously as providers without jeopardizing patient care.* Attendings criticized residents who gave up their autonomy by deferring to supervising doctors for decisions. The participants expected residents to rely less on preceptors as they advanced in their training. However, all participants understood that residents could encounter situations where they do not know what to do. In these cases, they expected residents to formulate plans and, if they are unsure how to proceed, ask their attending. Here is one example as an intern reflected, “*The patient was on two SSRIs! I think I was a little confused at that point. I was like, ‘why! [But] this is my first visit, who am I to question the doctor before me. But, this doesn’t seem right’. I went to go follow up with one of the attendings and get their opinion.’”* (R2).

Residents were criticized for not seeking instruction with complicated conditions and, instead, making decisions on their own. Without seeking support, the resident was left to mange patients alone, and at times, with substandard plans. In one example, the attending noted, “*She goes and shoots herself, and they find out she was just at the doctor’s office - ‘cause I’m sure the preceptor didn’t get this as, ‘Hey! She’s got major depression with suicidal ideation.’”* (A2).

## Discussion

This is the first study to synthesize the viewpoints of both attendings and residents regarding the norms of practice and the tensions between expected values in primary care residency training. Residents’ actions are better understood and judged from the participants’ perspectives and not the observers’. Participants’ remarks demonstrate that a resident’s action is judged to be adequate when it simultaneously upholds many values.

Prior research has focused on presenting the work of doctors as of technical skills that can be judged, often out of context, by an observer. Objectivated criteria have been suggested to assess performances. For example, Roter et al. imply that evidence of improved outcomes include the increased use of certain types of questions (open-ended, relationship-building statements, etc.) and letting patients talk more [7]. My study shows that residents’ performance must be judged in their context. An open-ended statement can be the right choice in a particular context and the wrong one in another. Letting patients just talk without guidance may not be inappropriate. Further, the person performing the act provides invaluable insights to complement what a conversant can provide of critique. Thus, the first practical recommendation is to *implement more dialogical and reflective approaches in residency training instead of relying solely on observation and checklists of performance criteria.* This recommendation is consistent with the work of Kumagai et al., who called for more spaces in the curriculum for reflection and for learning in dialogue [27]. It is also in line with empirical research showing the effectiveness of such approaches [28, 29].

Previous research has shown that less than errors occur while residents are training [30–32]. My study contributes to the understanding of the kinds of omissions residents make. Residents were criticized because they did not adequately perform some of the basic tasks of the visit, such as eliciting sufficient information from a suicidal patient, diagnosing the reason for a patient’s fracture not healing, or at times not giving the right advice. They also acknowledged that they, accidentally or consciously, did not address the patient’s concerns. Furthermore, at times they aimed to address too many problems and ended up with only a partially adequate job for a few. The most common theme behind these criticized practices, however, was that residents often did not know what they did not know. Residents are building competencies, but they are not always competent. My study showed that when faced with uncertainty, residents quite often made decisions without asking for support from a supervisor. Therefore, my second practical recommendation is to *advance the conversation on supervision strategies that directly involve a more competent other in the care of patients, even when residents are advanced in training.*

My study has many strengths. Unlike studies that relied on standardized patients or structured settings [11, 33–35], I used actual patient interactions in the natural context. When including the perspectives gained from the video recordings to allow for observation of precisely what took place, this research method provided depth in the data collection, especially with the triangulation of perspectives of different participants. However, the work is not without limitations. First, the study was done in one institution, and learning can only be transferrable with caution to other institutes with different cultures and norms. Second, while I believe the included patients’ sample is fairly diverse, as a naturally occurring experiment, other important patient representations may have been missed.

Future work will aim to explore how learning takes place over time when residents engage in a model of reflection and dialogue. I expect to see resident performance developing to better adhere to the explicated norms. I also anticipate seeing more robust critical reflections and dialogues on these norms and values.

## Conclusion

Participants in the conversations, both residents and attendings, addressed practice norms, and highlighted resident struggles around the tensions between different values. It should be a priority to implement dialogical and reflective approaches in residency training instead of relying solely on observation and feedback. Further conversations are necessary to develop supervision strategies that directly involve a more competent other in the care of patients, even when residents are advanced in training.

## Data Availability

The datasets used and/or analysed during the current study available from the corresponding author on reasonable request.
